# Sake lees extract improves hepatic lipid accumulation in high fat diet-fed mice

**DOI:** 10.1186/s12944-017-0501-y

**Published:** 2017-06-03

**Authors:** Hisako Kubo, Masato Hoshi, Takuya Matsumoto, Motoko Irie, Shin Oura, Hiroko Tsutsumi, Yoji Hata, Yasuko Yamamoto, Kuniaki Saito

**Affiliations:** 1 Human Health Sciences, Graduate School of Medicine and Faculty of Medicine, Kyoto University, 54 Kawaharacho, Shogoin, Sakyo-ku, Kyoto, 606-8507 Japan; 2 Department of Biochemical and Analytical Sciences, Fujita Health University Graduate School of Health Sciences, 1-98 Dengakugakubo, Kutsukakecho, Toyoake, Aichi 470-1192 Japan; 3 Research Institute, Gekkeikan Sake Co. Ltd., 247 Minamihamcho, Fushimi, Kyoto, 612-8385 Japan; 4 Department of Disease Control and Prevention, Fujita Health University Graduate School of Health Sciences, 1-98 Dengakugakubo, Kutsukakecho, Toyoake, Aichi 470-1192 Japan

**Keywords:** Sake lees extract, NAFLD, Lipid accumulation, Insulin resistance

## Abstract

**Background:**

Nonalcoholic fatty liver disease (NAFLD) is increasing worldwide as one of the leading causes of chronic liver disease. Sake lees (SL) are secondary products of sake manufacturing and are considered to have beneficial effects on human health. To investigate these effects, we used high fat diet (HFD)-fed mice treated with or without the SL extract.

**Method:**

Mice were the HFD ad libitum for 8 weeks and were administered 500 μL of distilled water with or without the SL extract (350 mg/mL) by a feeding needle daily for the last 4 weeks. Food intake, body weight, and liver weight were measured. Triacylglycerol content and the mRNA and protein expression levels of various lipid and glucose metabolism-related genes were determined in liver tissues. The levels of triglyceride, free fatty acids, glucose, insulin, and liver cell damage markers were determined in serum. Fatty acid-induced lipid accumulation in HepG2 cells was assessed in the presence or absence of the SL extract.

**Results:**

Mice fed a HFD and treated with the SL extract demonstrated a significant reduction in hepatic lipid accumulation and mRNA and protein levels of peroxidome proliferator-activated receptor γ (PPARγ), PPARα, CD36, and phosphoenolpyruvate carboxykinase 1 in the liver, while the SL extract did not affect body weight and food intake. Moreover, insulin resistance and hepatic inflammation in HFD-fed mice improved after administration of the SL extract. In HepG2 cells, the SL extract suppressed fatty acid-induced intracellular lipid accumulation.

**Conclusions:**

These findings suggest that treatment with the SL extract could potentially reduce the risk of NAFLD development, and that the SL extract may be clinically useful for the treatment of NAFLD.

## Background

Nonalcoholic fatty liver disease (NAFLD) is emerging as a major public health problem because of its association with increased cardiovascular and liver-related morbidity and mortality [1–4]. Both genetic factors and lifestyle, including dietary habits and physical activity, contribute to the pathogenesis of NAFLD. The primary therapeutic approach is to recommend healthy lifestyle strategies that focus on reducing body weight and increasing insulin sensitivity, including dietary and exercise regimens. Although these strategies are effective in randomized controlled trials, their impact on the incidence and severity of NAFLD at the population level is limited, due to poor patient compliance [2, 5, 6], as it is difficult for NAFLD patients to maintain a well-balanced and well-proportioned diet. Several anti-NAFLD agents are currently in the preclinical development stage. Additionally, metformin, statins, and fibrates, are currently being tested in clinical trials as treatments for NAFLD. However, these drugs have significant adverse side effects, including enhanced risk of infection and osteoporosis [7–9]. Thus, novel treatment candidates with high efficacy and fewer side effects are urgently needed for patients with NAFLD.

Functional foods that patients can easily eat on a daily basis may be useful in the treatment of NAFLD. Sake lees (SL) are secondary products of the sake manufacturing process. Sake is a traditional Japanese rice wine, and is used in condiments for making traditional Japanese foods such as soup. SL are thought to have beneficial effects on human health as it contains large proportions of protein, carbohydrates, and vitamins [10–12], and there is evidence to support its health benefits. For example, SL fermented with lactic acid bacteria prevent allergic rhinitis-like symptoms and IgE-mediated basophil degranulation [10]. SL and sake yeast contain functional food components, such as angiotensin І-converting enzyme inhibitory peptides, that have antihypertensive activity [11]. Furthermore, sake yeast has been shown to suppress alcohol-induced liver injury in mice [12]. In rats, the intake of a SL-rich diet increased spontaneous locomotive activity [13] and had a hypolipidemic effect with no harmful side-effects [14]. However, the effects of SL on high fat diet (HFD)-induced obesity in mice have not been fully explored.

In this study, we first investigated the effects of the SL extract on lipid and glucose metabolism, and discovered that the SL extract improves hepatic lipid accumulation and insulin resistance in the HFD-fed mice. We then demonstrated that daily intake of the SL extract could be a potentially effective and safe approach to attenuating hepatic lipid accumulation.

## Methods

### Sake lees extract

**Fig. 1:**
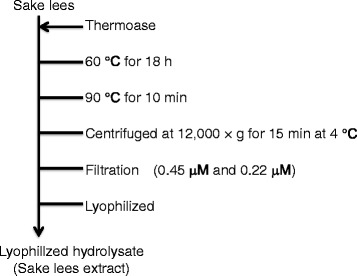
Purification procedure for the SL extract. The Sake lees (SL) were digested by protease at 60 °C for 18 h, filtrated and then lyophilized. Hydrolysis was performed so that the substrate concentration was 18% in water and the enzyme concentration was 0.2% of the substrate

Sake lees (SL) were produced using a liquefied product of rice slurry as the raw material (water content: 50%, crude protein: 35%, sugars: 8%, other: 7%). The SL were digested by protease Thermoase (Amano Enzyme Inc., Nagoya, Japan) at 60 °C for 18 h, filtrated and then lyophilized (Fig. 1). Hydrolysis was performed so that the substrate concentration was 18% in water and the enzyme concentration was 0.2% of the substrate.

### Mice

Six-week-old male C57BL/6 J mice were purchased from Japan SLC Inc. (Shizuoka, Japan). The mice were housed at 25 °C with a 12 h light, 12 h dark cycle. All experiments were performed in accordance with the Guidelines for Animal Care at Kyoto University. The mice were fed a normal diet (ND) containing 5% fat by weight (CE-2; CLEA Japan, Tokyo, Japan) and tap water for 1 week. Then, the mice were divided into five groups as follows: Mice in the first group were fed the ND ad libitum for 4 weeks (4 weeks’ ND group). Mice in the second group were fed ad libitum a high fat diet (HFD) 32% fat by weight (HFD32; CLEA Japan, Tokyo, Japan) for 4 weeks (4 weeks’ HFD group). Mice in the third group were fed the ND ad libitum for 8 weeks and administered 500 μL of distilled water without the SL extract using a feeding needle daily for the last 4 weeks (ND group). Mice in the fourth and the fifth group were the HFD ad libitum for 8 weeks and were administered 500 μL of distilled water with or without the SL extract (350 mg/mL) by a feeding needle daily for the last 4 weeks (SL extract group; HFD group, respectively). The amount of total food intake was measured at 8-week HFD feeding and body weight were measured at each week of HFD feeding. To obtain samples, the animals were anesthetized and humanely sacrificed at the end of the experimental period after fasting for approximately 18 h. Blood was collected from the abdominal vena cava and centrifuged at 3000 × *g* for 10 min at room temperature. Liver and epididymal white adipose tissue (WAT) were excised, washed in ice-cold 0.9% saline, wiped on filter paper, and then weighed. The obtained serum, liver, and epididymal WAT were stored at −80 °C until use.

### Glucose homeostasis

The oral glucose tolerance test (OGTT) and insulin tolerance test (ITT) at 8 week were performed by the methods described previously [15] with slight modifications regarding the amounts of glucose and insulin administered. In the OGTT, glucose (1.0 g/kg body weight (BW)) was orally administered after 6 h fasting. Blood glucose and insulin measurements were evaluated at 15, 30, 60, 120 min after administration. In the ITT, insulin (0.75 unit/kg BW) was intraperitoneally injected. Blood glucose measurements were evaluated at 15, 30 min after administration.

### Histopathology and hepatic lipid measurement

The livers were dissected and cryopreserved in OTC compound (Sakura Finetek Japan, Ltd., Tokyo, Japan) at −80 °C. They were sectioned (4 μm thick) with a Cryostat Leica CM1900 (Leica Microsystems K.K., Tokyo, Japan) and stained with Oil Red O. Sections were viewed with a light microscope CX41 (Olympus, Tokyo, Japan).

For hepatic lipid measurements, the homogenate of mouse liver (40–60 mg) was extracted with 1 mL of chloroform/methanol (2:1), vortexed for 15 min and then incubated overnight. The supernatant was vortexed with 0.5 mL of distilled water, incubated for 30 min, and then centrifuged at 7700×*g* for 10 min at 4 °C. The organic phase was collected, vortexed with 0.6 mL of chloroform/methanol/distilled water (2:1:3) for 30 min, incubated for 30 min, and then centrifuged at 7700×*g* for 10 min at 4 °C. The organic phase was collected, dried and resuspended in 1 mL of 2-propanol/Triton X-100 (19:1). Triacylglycerol (TG) and total cholesterol (T-CHO) levels were determined using the clinical biochemistry automatic analyzer BioMajesty JCA-BM 2250 (JEOL Ltd., Tokyo, Japan).

### Measurement of serum biochemical parameters

Serum T-CHO, glucose, aspartate transaminase (AST), and alanine transaminase (ALT) levels were determined using the automatic analyzer. Serum glycoalbumin, free fatty acid (FFA), and ketone bodies levels were determined using the Hitachi 7180 biochemistry automatic analyzer (Hitachi Ltd., Tokyo, Japan). Serum insulin levels were determined using an Insulin ELISA Kit (Shibayagi, Gunma, Japan). The homeostasis model assessment of insulin resistance (HOMA-IR) index was calculated using the following equation to assess insulin resistance [16]:

HOMA ‐ IR = fasting serum glucose (pmol/L) × fasting serum insulin (mmol/L)/22.5.

### Cells and culture

Human hepatoma cell line (HepG2) was obtained from the American Type Culture Collection (ATCC, Bethesda, MD, USA), and maintained in DMEM medium supplemented with 10% fetal bovine serum (FBS) and 100 U/mL of penicillin-streptomycin at 5% CO_2_. As described previously [17], HepG2 cells were seeded in wells of 6-well plates (1 × 10^6^/well), and were incubated with the medium for 24 h. The cells were then washed twice with phosphate-buffered saline, and the medium was changed to FBS-free DMEM containing 0.5% bovine serum albumin and antibiotics. After incubation for 1 h, the cells were treated with or without the SL extract (0.1 mg/mL or 1.0 mg/mL) and/or fatty acid mixture (100 μM linoleic acid and 100 μM oleic acid) (Sigma-Aldrich, St. Louis, MO, USA) for 18 h. After treatment, the cells were fixed with 10% formalin, stained with 60% Oil Red O, and the red-stained lipid droplets were observed under a light microscope. To quantify lipid accumulation, Oil Red O from the stained cells was eluted by adding 100% isopropanol and the optical density was detected using a spectrophotometer at 490 nm as previously described [18].

### RNA extraction and RT-PCR

The total RNA was extracted from mouse livers with ISOGEN (Nippon GENE, Tokyo, Japan) and the RNA concentration was determined spectrophotometrically at 260 nm. Reverse transcription-PCR (RT-PCR) was performed using a Revetra Ace Kit (TOYOBO, Osaka, Japan). Primers used in this study were shown as Table 1. For amplification reactions, the Go Taq Green Master Mix (Promega, Madison, WI, USA) was used according to the manufacturer’s instructions. The conditions for the reaction were: 30 s at 94 °C, 30 s at Tm (Table 1), and 30 s at 72 °C. The products were visualized by electrophoresis on 4% agarose gels. Semi-quantitative analysis of RT-PCR products was performed using NIH ImageJ 1.34 s software and normalized to β-actin.

**Table 1:** Primer sequences for RT-PCR

Gene	Primers	Sequence (5′-3′)	Size (bp)	Tm (°C)
β-actin	Sense	GGACTCCTATGTGGGTGACGAGG	366	55
	Antisense	GGGAGAGCATAGCCCTCGTAGAT		
Peroxisome proliferator-activated receptor γ (PPARγ)	Sense	TCCGTGATGGAAGACCACTCGCAT	124	55
	Antisense	CAGCAACCATTGGGTCAGCTCTTG		
Peroxisome proliferator-activated receptor α (PPARα)	Sense	TCTCCAGCTTCCAGCCCTTCCTCA	144	55
	Antisense	TTCACATGCGTGAACTCCGTAGTG		
Liver X receptor α (LXRα)	Sense	TCCATCAACCACCCCCACGAC	328	63
	Antisense	CAGCCAGAAAACACCCAACCT		
Sterol regulatory element-binding transcription factor 1 (SREBP-1c)	Sense	GTAGGTCACCGTTTCTTTGTGGAC	163	60
	Antisense	TGGGCTGAGCAATACAGTTCAAC		
CD36	Sense	GAACCACTGCTTTCAAAAACTGG	102	55
	Antisense	TGCTGTTCTTTGCCACGTCA		
Acetyl-CoA carboxylase (ACC)	Sense	AGGAGGGAAAGGGATCAGAA	435	57
	Antisense	TGTGCTGCAGGAAGATTGAC		
Fatty acid synthase (FAS)	Sense	TGGTGGTGTGGACATGGTCACAGA	160	60
	Antisense	CCGAAGCTGGGGGTCCATTGTGTG		
Carnitine palmitoyltransferase I (CPT1)	Sense	CCTGGGCATGATTGCAAAG	330	57
	Antisense	ACAGACTCCAGGTACCTGCTCA		
Phosphoenolpyruvate carboxykinase (PCK1)	Sense	CTCTGATCCAGACCTTCCAA	105	60
	Antisense	GAAGTCCAGACCGTTATGCAG		
Glucose 6-phosphatase (G6Pase)	Sense	GATTGCTGACCTGAGGAACGC	198	59
	Antisense	ATAGGCACGGAGCTGTTGCTG		
Glucokinase (GCK)	Sense	GTGTACAAGCTGCACCCGA	301	58
	Antisense	CAGCATGCAAGCCTTCTTG		
Phosphofructokinase (PFK)	Sense	CGATCTATCTACCTATGCCGACA	245	59
	Antisense	ACACCCGCATCAATCTCATTCA		

### Western blot

For the preparation of liver cytosol, frozen liver tissues were homogenized in the RIPA buffer (Wako Pure Chemical, Osaka, Japan) at 4 °C. The total protein concentrations of the liver tissue extract were determined by using DC protein assay (Bio Rad, Hercules, CA, USA). The proteins were separated by SDS-PAGE and were electrophoretically transferred onto PVDF membrane (Trans-Blot Turbo Blotting System; Bio Rad). The membranes were first incubated with the primary antibodies against peroxisome proliferator-activated receptor γ (PPARγ) (#5468; Cell Signaling Technology, Danvers, MA, USA), PPARα (GTX101098; Gene Tex, Irvine, CA, USA), CD36 (ab133625; Abcam, Cambridge, MA, USA), phosphoenolpyruvate carboxykinase 1 (PCK1) (#5105; Cell Signaling Technology), and β-actin (PM053; MBL, Nagoya, Japan). The membrane was then incubated with the horseradish peroxidase (HRP) -coupled secondary antibodies (for PPARγ and PPARα: 111–035-045; Jackson Immuno Research Laboratories, West Grove, PA, USA; for CD36, PCK1, and β-actin: ab6721; Abcam). Detection was performed with Immuno Star LD (Wako Pure Chemical). The protein bands were quantified by densitometry using the NIH ImageJ 1.34 s software and normalized to β-actin.

### Statistical analysis

All data are expressed as the mean ± SEM. Statistical differences between more than three groups were determined using one-way ANOVA followed by Dunnett’s test. Statistical analyses and graphs were performed using Graph Pad Prism version 5.0 (Graph Pad Software, San Diego, USA).

## Results

### The SL extract improves hepatic lipid accumulation in HFD-fed mice

**Fig. 2:**
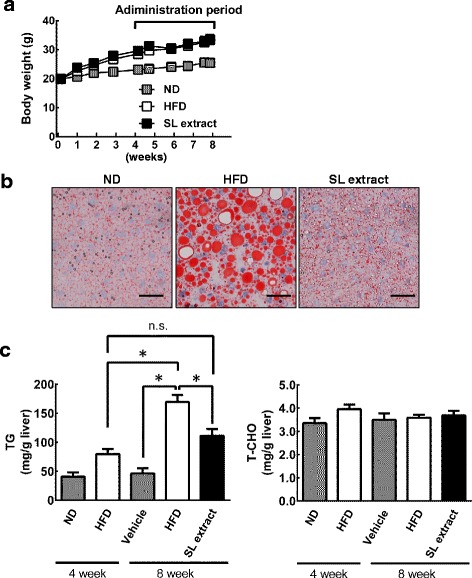
Treatment with the SL extract reduces hepatic lipid accumulation in HFD-fed mice. Mice were divided into five groups as follows: Mice in the first group were fed a normal diet (ND) for 4 weeks (4 weeks’ ND group). Mice in the second group were fed a high fat diet (HFD) for 4 weeks (4 weeks’ HFD group). Mice in the third group were fed the ND for 8 weeks and administered 500 μL of distilled water without the SL extract using a feeding needle daily for the last 4 weeks (ND group). Mice in the fourth and the fifth group were the HFD for 8 weeks and were administered 500 μL of distilled water with or without the SL extract (350 mg/mL) by a feeding needle daily for the last 4 weeks (SL extract group; HFD group, respectively). **a** The change in body weight in the ND, HFD, and SL extract groups during the feeding period of 8 weeks. There was no significant difference in body weight between the HFD group and the SL extract group. **b** The three groups were humanely sacrificed at 8 week. The treatment with the SL extract reduced the hepatic lipid accumulation in HFD-fed mice. Liver was stained by Oil Red O and visualized under a microscope. Scale bars indicate 50 μm. **c** The hepatic triglyceride (TG) levels in the SL extract group at 8 weeks were lower than those in HFD group at 8 weeks. On the other hand, there was no significant difference in the hepatic total-cholesterol (T-CHO) content between the five groups. Data are expressed as the mean ± SEM of five mice (4 weeks’ ND group, 4 weeks’ HFD group, and ND group), nine mice (HFD group) and nine mice (SL extract group). Statistically significant differences between groups were determined using ANOVA; *p < 0.001, n.s.: not significant

To elucidate the effects of the SL extract on HFD-induced lipid accumulation, we compared the lipid accumulation in the HFD group and the SL extract group. We assessed the changes in body weight for each group. Although there was no difference in body weight between the HFD group and the SL extract group (Fig. 2a), nor food intake (Table 2), the liver weight and lipid content were significantly lower in the SL extract group than in the HFD group. Oil Red O staining of the liver tissue sections obtained from the SL extract group showed the accumulation of HFD-induced lipid, but the formation of large lipid droplets in the liver was reduced (Fig. 2b). Moreover, the liver TG levels in the SL extract group at 8 weeks were lower than those in HFD group at 8 weeks, and were similar to those in the HFD group at 4 weeks (Fig. 2c). There was no significant difference in hepatic total-cholesterol (T-CHO) content between the five groups (Fig. 2c).

**Table 2:** Effects of the SL extract on body lipid accumulation and serum components in mice

	ND group	HFD group	SL extract group
Final body weight, g	25.5 ± 1.0^b^	33.4 ± 1.0	33.5 ± 1.8
4 week total food intake, g/mouse	85.8	119.6	125.3
8 week total food intake, g/mouse	159.5	204.7	216.6
Liver, g/100 g body weight	3.56 ± 0.12	3.69 ± 0.09	3.16 ± 0.19^a^
Epididymal WAT, g/100 g body weight	3.01 ± 0.15^c^	6.78 ± 0.56	6.22 ± 0.43
Serum glucose, mg/dL	43.0 ± 4.2^c^	173.0 ± 10.9	140.8 ± 2.6^a^
Serum Insulin, ng/mL	0.29 ± 0.07^b^	0.93 ± 0.14	0.46 ± 0.07^a^
HOMA-IR	0.33 ± 0.09^b^	2.14 ± 0.35	0.85 ± 0.13^b^
Serum glycoalbumin, %	3.7 ± 0.1^c^	4.5 ± 0.1	4.1 ± 0.1^b^
Serum T-CHO, mg/dL	77.0 ± 5.5^c^	169.8 ± 6.3	141.0 ± 4.7^b^
Serum TG, mg/dL	69.5 ± 8.7^a^	45.1 ± 7.6	39.8 ± 2.9
Serum FFA, mEq/L	1.03 ± 0.02^b^	0.82 ± 0.06	0.79 ± 0.03
Serum ketone bodies, mmol/L	2.03 ± 0.01	2.40 ± 0.14	3.18 ± 0.23^b^
Serum ALT, IU/L	17.5 ± 1.2^c^	87.1 ± 10.8	31.9 ± 5.9^c^
Serum AST, IU/L	49.5 ± 1.7^c^	146.6 ± 13.9	79.0 ± 8.3^c^

Data are expressed as the mean ± SEM of five mice (ND group), nine mice (HFD group and nine mice (SL extract group) except total food intake. Statistically significant differences between groups were determined using ANOVA; ^a^ *p* < 0.05,^b^ *p* < 0.01,^c^ *p* < 0.001 vs. the HFD group

**Fig. 3:**
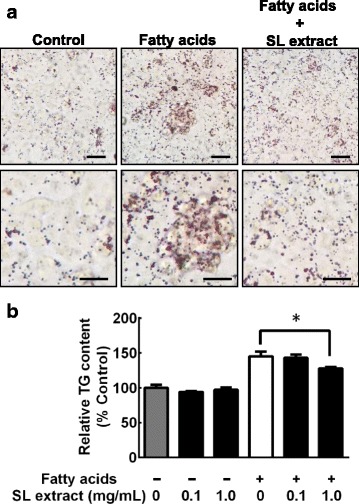
The SL extract suppresses lipid accumulation in HepG2 cells. HepG2 cells were treated with fatty acids (100 μM linoleic acid and 100 μM oleic acid) in the presence of or absence of the SL extract (0.1 or 1.0 mg/mL) for 18 h. **a** Lipid droplets were assessed by Oil Red O staining. The lipid droplets were increased by fatty acids, but the treatment with the SL extract suppressed the increase of intracellular TG. Scale bars indicate upper panel; 100 μm, lower panel; 50 μm. **b** To quantify lipid accumulation, Oil Red O from the stained cells was eluted by adding 100% isopropanol and the optical density was detected using a spectrophotometer at 490 nm. Data are expressed as the mean ± SEM from three independent experiments. Statistically significant differences between groups were determined using ANOVA; *p < 0.05

To examine whether the effects of the SL extract were the results of a direct or an indirect pathway, we treated HepG2 human hepatoma cells with fatty acids in the presence of or absence of the SL extract to evaluate intracellular lipid accumulation in vitro (Fig. 3a and b). The intracellular TG level of the cells treated with fatty acids in the presence of the SL extract was significantly lower than the level in the cells treated with fatty acids only. These results suggest that treatment with the SL extract directly improved hepatic steatosis via suppression of fatty acid-induced intracellular lipid accumulation.

### The SL extract suppresses insulin resistance and hepatitis in HFD-fed mice

The levels of fasting serum glucose, insulin, HOMA-IR, and glycoalbumin were significantly higher in the HFD group than in the ND group (Table 2). These levels were significantly lower in the SL extract group than in the HFD group. To assess insulin secretion, hepatic insulin sensitivity and glucose metabolism on the whole body, we next performed OGTT and ITT (data not shown). On OGTT, blood glucose at 30 min after administrated in the SL extract group and in the HFD group were 438.3 ± 107.4 ng/mL and 479.2 ± 61.8 ng/mL, respectively. Blood insulin at 15 min after administrated tended to be lower in the SL extract group (2.35 ± 1.21 ng/mL) than in the HFD group (4.05 ± 3.48 ng/mL). On ITT, blood glucose at 40 min after administrated in the SL extract group and in the HFD group were 141.2 ± 37.0 ng/mL and 143.6 ± 11.8 ng/mL, respectively.

Serum ALT and AST activities were significantly higher in the HFD group than in the ND group but the enzyme activities in the SL group were significantly lower in than those in the HFD group (Table 2). Serum ketone bodies in the SL extract group demonstrated a significant increase compared to that in the HFD group (Table 2).

### The SL extract significantly regulates the expression of various lipid and glucose metabolism-related genes in the liver

**Fig. 4:**
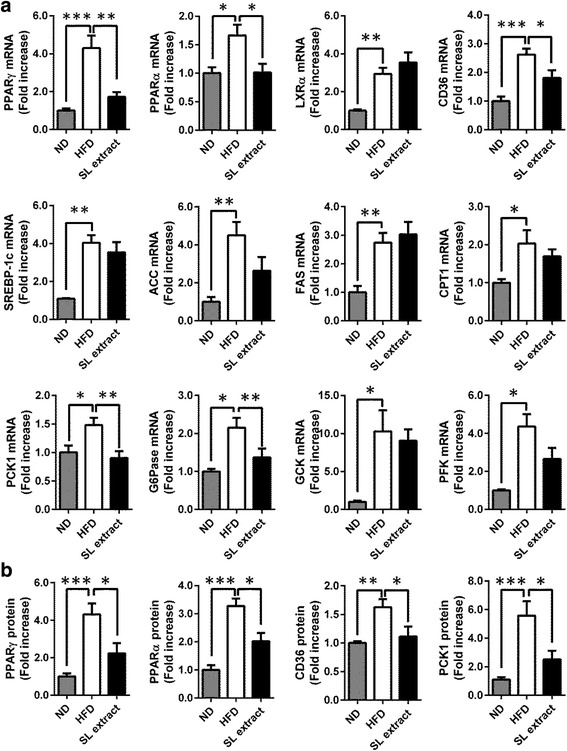
The SL extract decreases the mRNA levels of lipid and glucose metabolism related-factor in the liver. The three groups were humanely sacrificed at 8 week. The levels of mRNA (**a**) and protein levels (**b**) in the liver from each group of mice were determined by using RT-PCR and Western blot. Each mRNA or protein was normalized to β-actin mRNA or protein levels, and data are expressed with the value for the ND group defined as 1. **a** The mRNA levels of peroxisome proliferator-activated receptor γ (PPARγ), CD36, phosphoenolpyruvate carboxykinase 1 (PCK1), and glucose 6-phosphatase (G6Pase) in the liver were significantly suppressed by treatment with the SL extract. Data are expressed as the mean ± SEM of five mice (ND group), nine mice (HFD group) and nine mice (SL extract group). **b** The protein levels of PPARγ, CD36, PPARα, and PCK1 in the liver were significantly suppressed by treatment with the SL extract. Data are expressed as the mean ± SEM of five mice (each group). Statistically significant differences between groups were determined using ANOVA; *p < 0.05, **p < 0.01, ***p < 0.001 vs. the HFD group

To elucidate the underlying mechanisms of the effects of the SL extract, we measured the mRNA expression of various genes associated with lipid and glucose metabolism (Fig. 4a). The levels of PPARγ, PPARα, liver X receptor α (LXRα), CD36, and sterol regulatory element-binding transcription factor 1 (SREBP-1c), which function as the regulators of lipid metabolism, were significantly higher in the liver from the HFD group than in the liver from the ND group. The levels of PPARγ, PPARα, and CD36 in the liver from the SL extract group were significantly lower in the liver from the HFD group, although there were no differences in the levels of LXRα and SREBP-1c between the two groups. The levels of acetyl-CoA carboxylase (ACC), fatty acid synthase (FAS), and carnitine palmitoyltransferase I (CPT1), which participate in lipid metabolism, were significantly higher in the liver from the HFD group than in the liver from the ND group. However, there were no significant differences in these levels between the HFD and SL extract groups. The levels of PCK1, Glucose 6-phosphatase (G6Pase), glucokinase (GCK), and phosphofructokinase (PFK), which participate in glucose metabolism, were significantly higher in the liver from the HFD group than in the liver from the ND group. The levels of PCK1 and G6Pase, as markers of gluconeogenesis, were significantly lower in the liver from the SL extract group than in the liver from the HFD group.

The protein levels of PPARγ, PPARα CD36, and PCK1 were significantly higher in the liver from the HFD group than in the liver from the ND group and these protein expression levels were significantly lower in the SL extract group than in the HFD group (Fig. 4b).

## Discussion

In this study, we demonstrated that treatment with the SL extract ameliorates hepatic lipid accumulation and insulin resistance in HFD-fed mice via suppressing fatty acid-induced intracellular lipid accumulation by regulating lipid and glucose metabolism-related molecules.

Treatment with the SL extract significantly ameliorated hepatic TG accumulation in the HFD group. The liver weight and lipid content of the SL extract group were significantly lower than those in the HFD group. However, there was no difference in body weight between the HFD group and the SL extract group, nor food intake. We speculate that there may be significant difference in body weight and adipose tissues between the HFD group and the SL extract group, if the SL extract administration period is more than 4 weeks. Indeed, treatment with other extracts for more than 4 weeks has the effect on body weight reduction [19, 20]. Treatment with the SL extract could prove to be a beneficial therapy for NAFLD without sudden weight loss. A previous study demonstrated that weight loss is a poor marker for visceral adipose tissue change [21]. Whereas dietary restriction has superior effects on body weight reduction compared to exercise [22, 23], exercise tends to have superior effects in reducing visceral adipose tissue compared to caloric restriction [24]. The reduction of visceral adipose tissue is more important in the treatment of NAFLD than body weight loss. The anti-NAFLD effect of the SL extract administration may be more prominent with the addition of exercise.

Treatment with the SL extract improved serum glucose, insulin, and hepatic insulin resistance in HFD-fed mice. Furthermore, our study found that SL extract administration had a suppressive effect on hepatic lipid accumulation by a direct pathway. In patients with NAFLD, insulin cannot suppress gluconeogenesis and does not convert glucose to glycogen after meals, a condition known as hepatic insulin resistance. Previous studies found that the excessive hepatic accumulation of TG and FFA induced hepatic insulin resistance [25, 26]. Therefore, the suppression of hepatic TG accumulation by treatment with the SL extract resulted in reduced hepatic insulin resistance. In NAFLD pathogenesis, imbalanced lipid metabolism and insulin resistance lead to simple steatosis and renders hepatocytes susceptible to adipocytokine imbalance, oxidative damage, dysregulated hepatocyte apoptosis, and activation of pro-fibrogenic factors and pro-inflammatory mediators [27]. Inflammation has been shown to be involved in the developmental process of fatty liver disease. Treatment with the SL extract suppressed serum ALT and AST elevations that were induced by the HFD. This result showed that treatment with the SL extract improved hepatic function and hepatitis.

The elevation of PPARγ mRNA and protein in the SL extract group were significantly suppressed, compared with the HFD group. It has been known that the main role of PPARγ in the liver is related to regulation of glucose and lipid metabolism [28, 29]. PPARγ expression is increased in the liver of obese patients and animals [30, 31]. In liver-specific PPARγ-deficient mice, the development of HFD-induced NAFLD and insulin resistance was suppressed [32]. Liver PPARγ regulates fatty acid uptake, and trafficking, as well as TG biosynthesis, which contributes to hepatic steatosis [33] and demonstrates positive associations with serum insulin levels and HOMA-IR and negative correlations with total adiponectin [30]. In contrast, several reports demonstrate that PPARγ agonists inhibited ectopic lipid accumulation and inflammation in the peripheral tissues and increased insulin sensitivity by converting hypertrophic adipocytes to normal adipocytes [15, 34]. Additionally, CD36 mainly functions in fatty acid uptake and the expression is transcriptionally regulated by PPARγ [35]. Hepatocyte-specific disruption of CD36 attenuates fatty liver disease and improves insulin sensitivity in HFD-fed mice [36]. It has been demonstrated that the expression of PPARα, the master regulator of fatty acid β-oxidation, is reduced in patients with NAFLD and HFD-fed mice [37–39]. However, we and other researcher have been reported that PPARα mRNA and protein levels were increased in the liver from the HFD group, compared with that in the liver from the ND group [40], and showed that serum FFA in the HFD group was lower than in the ND group. FFA is the PPARα ligand and the activation of PPARα by FFA promotes hepatic fatty acid to generate ketone bodies, providing an energy source for peripheral tissue [41]. Our data demonstrated that serum ketone bodies in the SL extract group were higher than in the HFD group. Thus, the treatment with SL extract may elevate fatty acid β-oxidation in the liver of HFD-fed mice under the reduction of the enhanced expression of PPARα mRNA and protein in the tissue. Additional studies are needed to determine the detailed mechanism about PPARα. In this study, the SL extract suppressed the expression of CD36 in the liver in HFD mice, associated with a decrease of PPARα and PPARγ. It suggested that the hepatic uptake of FA was inhibited by the effect of the SL extract in a high fat dietary condition. However, treatment with the SL extract made no significant differences in the expression of SREBP1c, which are involved in intrinsic FA production, in HFD mice. Therefore, our study identified that the SL extract could not influence intrinsic FA production though SREBP1c although the SL extract could suppress the hepatic uptake of FA by the down-regulation of PPARs in a fat-rich condition. It is the interesting evidences that explain the improvement of NAFLD induced by the SL, regarding the hepatic FA metabolism.

Moreover, PCK1 is a main control point for the regulation of gluconeogenesis and is necessary for the integration of hepatic energy metabolism [42]. Treatment with the SL extract improved hepatic gluconeogenesis in HFD-fed mice. The levels of serum glucose, insulin, HOMA-IR, and glycoalbumin were significantly higher in the HFD group than in the ND group. Treatment with the SL extract group tended to decrease blood insulin on OGTT, compared with those in the HFD group. However, there was no significant difference of OGTT and ITT between in the HFD group with in the SL extract group. These data showed that treatment with the SL extract slightly improved insulin secretion, hepatic insulin sensitivity, and glucose metabolism on the whole body. We speculate that there may be significant difference in glucose and insulin on OGTT and ITT between the HFD group and the SL extract group, if the SL extract administration period is more than 4 weeks.

Our study demonstrates that treatment with the SL extract suppresses the increased mRNA expression of PPARγ, PPARα, CD36, and PCK1 in HFD-fed mice. Thus, these results suggested that the SL extract affects the lipid and glucose metabolism. However, there is a possibility that the SL extract administration improves hepatic lipid accumulation in HFD-fed mice by affecting the other pathway. Hepatic energy metabolism is largely controlled at the genomic level by numerous transcription factors and co-regulators [43].

## Conclusions

The findings of the present study suggested that SL extract intake suppresses hepatic lipid accumulation and insulin resistance, and could potentially reduce the risk of developing NAFLD. We believe that the SL extract will be clinically useful for the treatment of NAFLD.

## Abbreviations

ACC: Acetyl-CoA carboxylase
ALT: Alanine transaminase
AST: Aspartate transaminase
CPT1: Carnitine palmitoyltransferase I
FAS: Fatty acid synthase
G6Pase: Glucose 6-phosphatase
GCK: Glucokinase
HFD: High fat diet
HOMA-IR: Homeostasis model assessment of insulin resistance
LXRα: Liver X receptor α
NAFLD: Nonalcoholic fatty liver disease
ND: Normal diet
PCK1: Phosphoenolpyruvate carboxykinase 1
PFK: Phosphofructokinase
PPARγ: Peroxisome proliferator-activated receptor γ
SL: Sake lees
SREBP-1c: Sterol regulatory element-binding transcription factor 1
T-CHO: Total cholesterol
TG: Triacylglycerol
